# A novel splice site variant c.1183 + 1 G > C in DFNA5 causing autosomal dominant nonsyndromic hearing loss in a Chinese family

**DOI:** 10.1186/s12920-022-01315-8

**Published:** 2022-07-21

**Authors:** Qiong Li, Shujuan Wang, Pengfei Liang, Wei Li, Jian Wang, Bei Fan, Yang Yang, Xiaogang An, Jun Chen, Dingjun Zha

**Affiliations:** Department of Otolaryngology-Head and Neck Surgery, Xijing Hospital, Air Force Medical University, 127 Changle West Road, Xi‘an, 710032 Shaanxi People’s Republic of China

**Keywords:** DFNA5, Exon skipping, *GSDME*, Nonsyndromic hearing loss, RNA splicing

## Abstract

**Background:**

The most frequent clinical presentation of autosomal dominant nonsyndromic hearing loss (ADNSHL) is bilateral, symmetrical, postlingual progressive sensorineural hearing loss, which begins with impairment at high frequencies and eventually progresses to hearing loss at all frequencies. Autosomal dominant deafness-5 (DFNA5) is a subtype of ADNSHL caused by heterozygous variants in the gasdermin E (*GSDME*, also known as DFNA5) gene.

**Methods:**

Deafness gene NGS panel analysis were performed on the proband of a six-generation Chinese family with hearing loss. The co-segregation analysis between the hearing loss and the novel variant was analyzed by Sanger sequencing and pure-tone audiometry. The minigene splicing assay was performed to evaluate the potential effect of the variant on messenger RNA splicing in vitro.

**Results:**

The family exhibited autosomal dominant, progressive, postlingual, nonsyndromic sensorineural hearing loss, which was similar to that of the previously reported DFNA5 families. A novel heterozygous splice site variant in *GSDME* gene intron 8 was identified, which co-segregated with the hearing loss phenotype of the family. The variant caused skipping of exon 8 in the mutant transcript, leading to the direct linking of exons 7 and 9.

**Conclusions:**

We identified a novel *GSDME* splice site variant c.1183 + 1 G > C in an extended Chinese family, which led to the skipping of exon 8. The results extended the pathogenic variants spectrum of the *GSDME* gene, provided further support for the 'gain-of-function' mechanism of DFNA5, and afforded a molecular interpretation for these patients with ADNSHL.

**Supplementary Information:**

The online version contains supplementary material available at 10.1186/s12920-022-01315-8.

## Introduction

Hearing loss is one of the most common congenital sensory defects in humans. Estimates show 1–3 hearing-impaired children in every 1000 newborns [1]. More than 60% of hearing loss was attributable to genetic factors [2, 3]. Based on the presence or absence of distinctive clinical features other than hearing loss, hereditary hearing loss can be categorized as follows: syndromic hearing loss and nonsyndromic hearing loss. Nonsyndromic hearing loss can be subdivided into autosomal dominant (DFNA), autosomal recessive (DFNB), X-linked (DFNX), and mitochondrial inheritance depending on the mode of inheritance. A total of 51 DFNA, 78 DFNB, 5 DFNX, and 2 mitochondrial inheritance have been identified to date (http://hereditaryhearingloss.org; accessed June 26, 2022). ADNSHL accounts for about 20% of nonsyndromic deafness and has a high degree of genetic heterogeneity, with different gene variants causing the same or similar phenotypes [4]. Genetic diagnosis is an important approach to discover the cause of nonsyndromic deafness because of the genetic heterogeneity.

DFNA5 (Phenotype MIM [5, 6]: 600,994) is a subtype of ADNSHL caused by heterozygous variants in the *GSDME* (Gene/Locus MIM: 608,798) gene. In 1995, *GSDME* was first mapped to 7p15 by Van Camp et al. using linkage analysis in an extended Dutch family. In 1998, *GSDME* was first cloned and identified as a deafness gene [7]. The *GSDME* gene including 10 exons, encodes a polypeptide of 496 amino acids with a mass of about 55 kDa. The clinical presentation associated with DFNA5 variants is the most commonly observed with ADNSHL, bilateral, symmetrical, high-frequency sensorineural hearing loss with postlingual onset, and further progression to all frequencies.

To date, more than a dozen DFNA5 families have been reported worldwide (Table 2). Although the pathogenic variants in *GSDME* of these families were different, these variants all caused the skipping of *GSDME* exon 8, led to a frameshift that changes amino acid residues 331 to 371, produced a premature stop codon at position 372 and resulted in the loss of 125 wildtype amino acids, formed a truncated protein and lost the normal carboxyl-terminal fragment [8]. This study, we aimed to report a novel c.1183 + 1 G > C splice site variant in the *GSDME* gene.

## Materials and methods

### Family recruitment and clinical evaluations

Due to progressive hearing loss that began a decade ago, the proband which was a 30-year-old man with family history of hearing loss visited the Department of Otolaryngology, Head and Neck Surgery, Xijing Hospital in 2020. Written informed consent was obtained from the proband and each participant of his family, and the study was approved by the Medical Ethics Committee of the First Affiliated Hospital of the Air Force Medical University (approval number KY20212002-C-1).

The following information was obtained from each study participant: identity information, age of onset, disease progression, mother's pregnancy, study participant's delivery, noise exposure, ototoxic drug use, head trauma, infectious diseases, family history, and other relevant clinical manifestations. Nongenetic causes of hearing loss, such as noise exposure, ototoxic drugs, and head trauma, were excluded. Physical examinations suggested no syndromic deafness in the family. Audiometric evaluations and otological examinations were performed for the proband, including pure-tone audiometry (PTA), acoustic impedance, distortion-product otoacoustic emission (DPOAE), auditory brainstem response (ABR), and temporal bone computed tomography. The other family members were evaluated using PTA. The average values of the thresholds of air conduction were determined at 500, 1000, 2000, and 4000 Hz to determine the degree of hearing loss in the family. The condition of an individual's hearing loss was classified as mild (26–40 dB HL), moderate (41–60 dB HL), severe (60–80 dB HL), or profound hearing loss (≥ 81 dB HL).

### Deafness gene NGS panel analysis

First, genomic DNA from members of this family was extracted from the peripheral blood leukocytes using a blood DNA extraction kit (cat. no. CW0544M, Kang Wei Century Blood Genome Non-column Extraction Kit). Second, the DNA library was prepared using a Standard Library Building Kit (MyGenostics GenCap Enrichment Technologies), including DNA fragmentation, end repair, adapter ligation, polymerase chain reaction (PCR) enrichment and product purification. Third, the qualified library was captured using a GenCap Deafness Capture Kit (MyGenostics GenCap Enrichment Technologies) and sequenced on an Illumina HiSeq X Ten sequencer. The DNA probes were designed to tile along the exon regions of the deafness genes. Subsequently, the raw data obtained by sequencing were saved in FASTQ format, followed by the bioinformatics analysis. The Cutadapt (https://cutadapt.readthedocs.io/en/stable/) was used to preprocess data to remove low-quality reads by a quality score ≥ 20. The clean reads were aligned to the UCSC hg19 human reference genome using BWA [9] (http://bio-bwa.sourceforge.net/). Duplicated reads were removed using Picard (http://broadinstitute.github.io/picard/) tools, and mapping reads were used for variant detection. The variants of SNP and InDel were detected and filtered using GATK [10] (https://www.broadinstitute.org/gatk/). The variants were further annotated using ANNOVAR [11] (http://annovar.openbioinformatics.org/en/latest/) and associated with multiple databases, such as 1000 Genomes database [12] (http://browser.1000genomes.org/), ESP6500 (http://evs.gs.washington.edu/EVS/), dbSNP (https://www.ncbi.nlm.nih.gov/snp/), EXAC (http://exac.broadinstitute.org), gnomAD (http://gnomad-sg.org) and HGMD (http://www.hgmd.cf.ac.uk/). The harmful effects of non-synonymous variants were evaluated using the four algorithms, SIFT (http://sift.jcvi.org/), PolyPhen-2 (http://genetics.bwh.harvard.edu/pph2/), MutationTaster (http://www.mutationtaster.org/), and GERP +  + (http://mendel.stanford.edu/SidowLab/downloads/gerp/index.html). Finally, the potential pathogenic variants were filtered based on the monogenic autosomal dominant trait. In the order of ‘heterozygosis, the frequency in the general population databases ≤ 0.02, located in exons or splicing sites, associated with autosomal dominant deafness’, the variants were filtered sequentially.

### Co-segregation analysis

Following deafness gene NGS panel analysis, the segregation analysis of candidate variant was completed by PCR and Sanger sequencing. A *GSDME* gene fragment was amplified and sequenced with the primers 5'-TTCTTCTTCCCTGCCCTACA-3' and 5’-CTCTGTGTCCCCAGAAGCAT-3’. PCR was performed with 25 µL reaction mixtures containing 100 ng genomic DNA, 1 µL of the forward and reverse primers, and 22 µL of 1.1 × Golden Star T6 Super PCR Mix (cat. no. TSE101, TsingKe Biological Technology). Thermocycling was performed using the following program: initial denaturation at 98 °C for 2 min, followed by 30 cycles of 98 °C for 10 s, 62 °C for 10 s, and 72 °C for 10 s, and then final extension at 72 °C for 1 min. The PCR products were purified using a Cycle Pure Kit (cat. no. D6492 OMEGA Bio‑Tek) and was mixed with sequencing primer and sequencing enzyme into a 5 µL system for reaction in thermocycler. The products were further purified and sequenced using the Applied Biosystems 3730 DNA Analyzer (Thermo Fisher Scientific, Inc.) to obtain sequencing data. All sequencing chromatograms were compared to the published sequence for *GSDME* (NM_004403).

### Evolutionary conservation analysis

The target sequence for alignment contained amino acid residues encoded by exon 7–exon 10. Multiple sequence alignment was performed across 18 species using BLAT on the UCSC Genome Browser (https://genome.ucsc.edu).

### Minigene splicing assay

A minigene splicing assay was performed to verify whether the variant affected splicing products. A partial sequence of the wild type and mutant type *GSDME*, including partial intron 6, exon 7, intron 7, exon 8, intron 8, exon 9, and partial intron 9 (5605 base pairs), was PCR amplified with gene-specific primers (F: TTATGGGGTACGGGATCACCAGAATTCgcatcgcagtcatgagactt and R: CGGGATCACCAGATATCTGGGATCCtggatgtctaccccctcatc) containing *Eco*RI and *Bam*HI restriction enzyme sites. PCR was performed with 50 µL reaction mixtures containing 200 ng DNA, 2 µL of the forward and reverse primers, and 44 µL of 1.1 × Golden Star T6 Super PCR Mix (cat. no. TSE101, TsingKe Biological Technology). The program for thermocycling is the same as above. After restriction enzyme digestion by *Eco*RI and *Bam*HI, the PCR fragment was ligated into the pSPL3 vector and sequenced. PCR and Sanger sequencing were used to evaluate whether the wild type and mutant type expression vectors were successfully constructed. The wild type or mutant type *GSDME* minigenes were transfected into COS7 cells (the kidney cells of the African green monkey) via lipofectamine. The cells were harvested 36–48 h after transfection. All cellular RNAs were extracted by AllPrep DNA/RNA/miRNA Universal Kit (cat. no. 80224, QIAGEN, Hilden, Germany) and used to produce cDNA by QuantiTect Rev. Transcription Kit (cat. no. 205311, QIAGEN, Hilden, Germany). The cDNA was PCR amplified with primer SD6-F (TCTGAGTCACCTGGACAACC) and SA2-R (ATCTCAGTGGTATTTGTGAGC). The PCR products were isolated after electrophoresis using 1.5% agarose gels and the gel bands were excised from the gel by TIANgel Midi Purification Kit (cat. no. DP209, TIANGEN, Beijing, China). The purified DNA verified by Sanger sequencing.

## Results

### Clinical characteristics

**Fig. 1:**
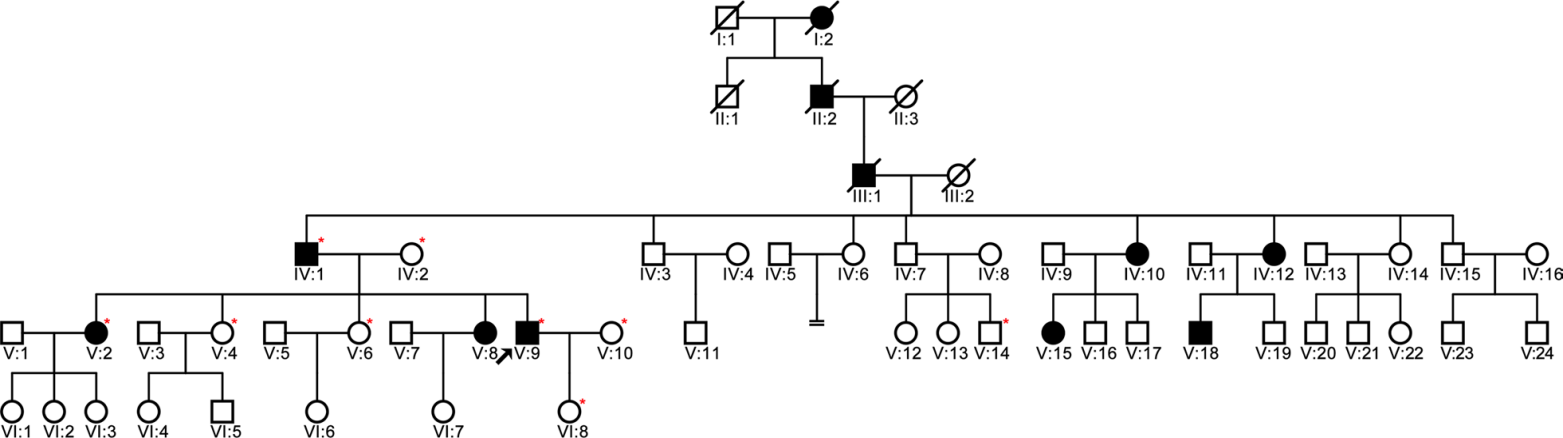
Family pedigree with autosomal dominant hearing loss. The affected individuals are denoted by filled symbols, and the unaffected individuals are denoted by an open symbol; the arrow indicates the proband (V:9); the red asterisks indicate the study participants

**Fig. 2:**
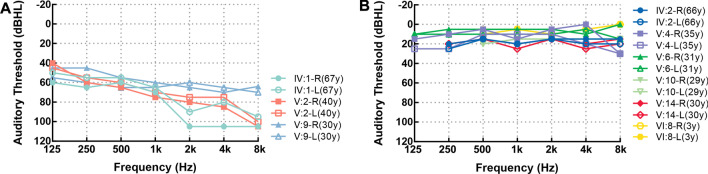
Pure‑tone audiograms of the family members. **A** Affected individuals IV:1, V:2, and V:9. **B** Unaffected individuals IV:2, V:4, V:6, V:10, V:14, and VI:8. R, Right; L, Left; y, years

The family contained 55 members in six-generations and had autosomal dominant, progressive, postlingual, nonsyndromic sensorineural hearing loss, and these features were consistent with ADNSHL (shown in Fig. 1). Nine members of this family participated in the present study, which included three affected and six unaffected relatives. The selected part of this family had 11 individuals with hearing loss, 3 of whom (IV:1, V:2, and V:9) were still alive and willing to join this study. The information regarding the mode of inheritance was obtained through the segregation/pedigree analysis. The information regarding the type of hearing loss was obtained by anamneses and clinical data. Pure‑tone audiograms of the three patients showed bilateral severe-to-profound sensorineural hearing loss (shown in Fig. 2A). Their hearing had begun to decline gradually at the age of 20 years, as mild loss, which then progressed to a severe loss. None of them have tinnitus, or vertigo, balance disorders, or symptoms involving other vestibular systems. The three individuals were all diagnosed with ADNSHL. The remaining family members were unaffected relatives; of these, six members (IV:2, V:4, V:6, V:10, V:14, and VI:8) underwent PTA, and their hearing was normal (shown in Fig. 2B and Table 1). The physical examination of all individuals in this family was otherwise unremarkable.

**Table 1:** Phenotype and genotype of individual family members

Family member	Age (year)	Nucleotide change	PTA-Right (dB HL)	PTA-Left (dB HL)	Age of onset (years)	Noise exposure	Ototoxic drugs	Head trauma
IV:1	67	c.1183 + 1 G > C	85	72.5	25	No	No	No
IV:2	66	Wild type	16.25	17.5	/	No	No	No
V:2	40	c.1183 + 1 G > C	76.25	70	18	No	No	No
V:4	35	Wild type	6.25	12.5	/	No	No	No
V:6	31	Wild type	6.25	8.75	/	No	No	No
V:9	30	c.1183 + 1 G > C	62.5	63.75	20	No	No	No
V:10	29	Wild type	16.25	16.25	/	No	No	No
V:14	30	Wild type	17.5	20	/	No	No	No
VI:8	3	Wild type	7.5	6.25	/	No	No	No

### Identification of the splice site variant (c.1183 + 1 G > C)

The genomic DNA of individual V:9 was subjected to deafness gene NGS panel analysis, including 406 deafness genes (shown in Additional file 1: Supplementary Table 1). The coverage of the targeted regions was 98.97% for the ≥ 10 × reads and 98.53% for the ≥ 20 × reads, respectively. The mean sequencing depth is 772.18 × . A total of 3255 variants were presented in Raw data (shown in Additional file 2: Supplementary Table 2), among the 2073 variants that were in heterozygosis, 241 variants had low frequency in the general population databases, 40 variants were located in exons or splicing sites, 3 variants may be associated with autosomal dominant deafness (shown in Additional file 3: Supplementary Table 3), 2 variants in the *WFS1* gene didn't segregated with the affected status in this family (shown in Additional file 1: Supplementary Fig. 3). Only a novel potentially splice site variant c.1183 + 1 G > C in the *GSDME* gene was likely to be pathogenic, according to the autosomal dominant inheritance pattern of the family. Further, the variant was neither reported in the literature nor present in database, such as The Human Gene Mutation Database (www.hgmd.cf.ac.uk; accessed June 26, 2022), Clinvar database (https://www.ncbi.nlm.nih.gov/clinvar/; accessed June 26, 2022), Leiden Open Variation Database (https://www.lovd.nl; accessed June 26, 2022) and Deafness Variation Databases (https://deafnessvariationdatabase.org; accessed June 26, 2022). The c.1183 + 1 G > C splice site variant in the *GSDME* gene was a novel potentially causative variant first discovered in this study.

### Co-segregation of genotype and phenotype

**Fig. 3:**
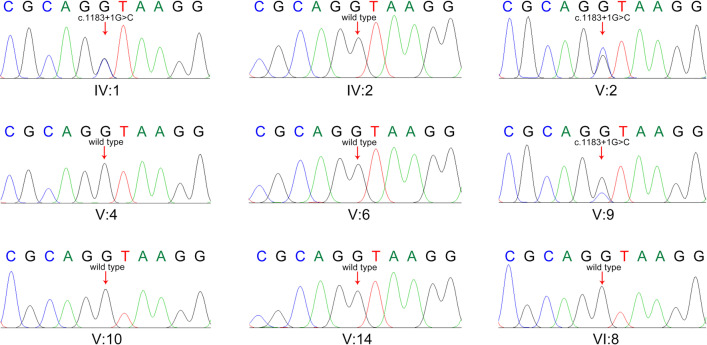
Sanger sequencing chromatograms of the variant c.1183 + 1 G > C from each study participant. Arrows indicate the position of the nucleotide changes identified in this study. IV:1, V:2, and V:9 carried the variant. The variant was not detected in individuals IV:2, V:4, V:6, V:10, V:14, and VI:8

Sanger sequencing was performed to confirm whether the c.1183 + 1 G > C splice site variant in the *GSDME* gene segregated with the affected status in this family. This variant was detected in individuals IV:1, V:2, and IV:9, and all these family members were diagnosed with progressive hearing loss. The variant was not detected in individuals IV:2, V:4, III:14, V:10, V:14, and VI:8, who had a normal hearing state (shown in Fig. 3). The Sanger sequencing result of all individuals in this family showed that this splice site variant precisely co-segregated with the progressive hearing loss phenotype in this family (Table 1).

### Functional characterization of the c.1183 + 1 G > C splice site variant on *GSDME* in vitro

**Fig. 4:**
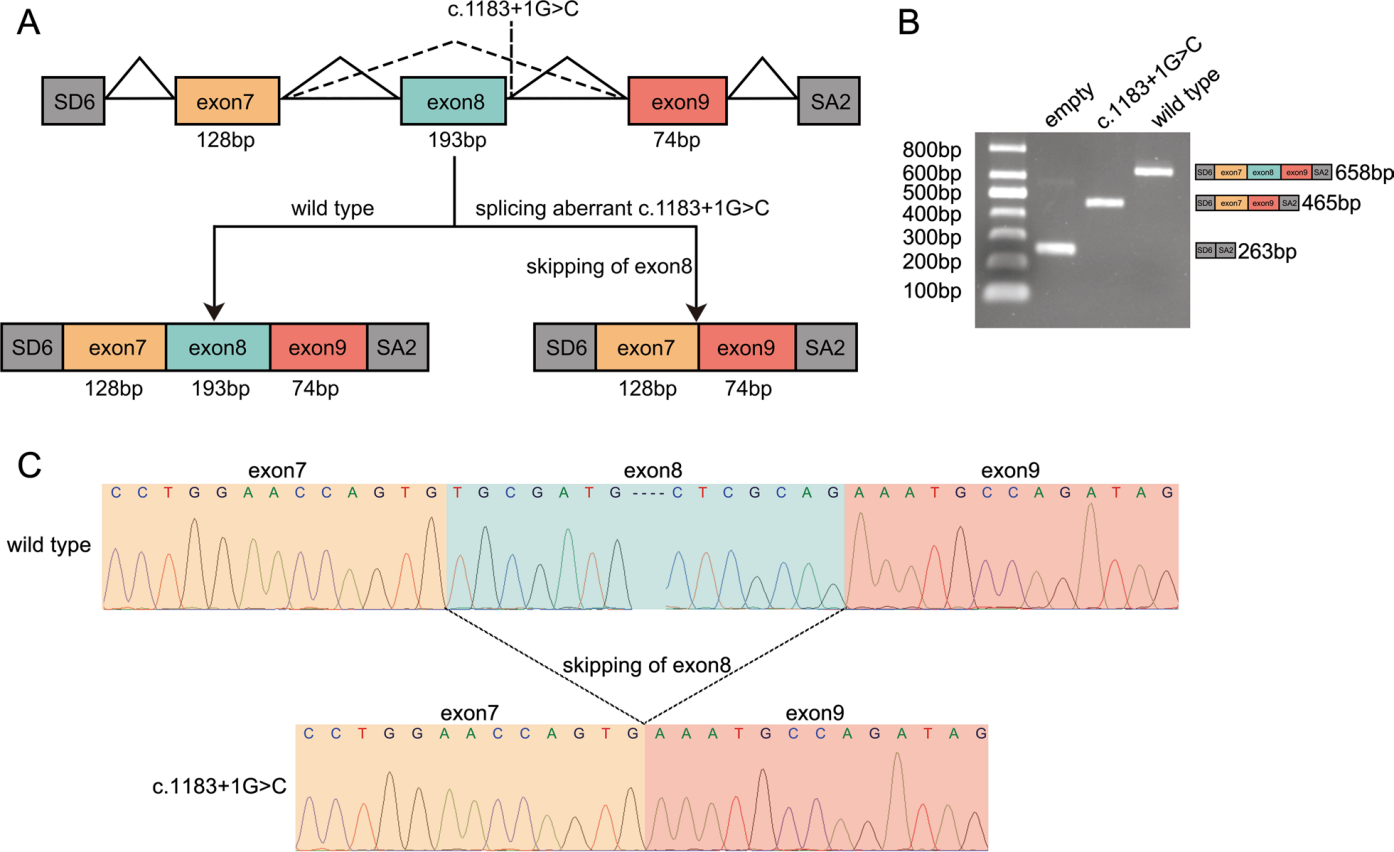
Identification of the splice site variant using the minigene splicing assay. **A** A schematic of the pSPL3 vector with cloned wild type and mutant type *GSDME*, including partial intron 6, exon 7 (yellow), intron 7, exon 8 (green), intron 8, exon 9 (red), and partial intron 9. A schematic of the splice products with the wild type splicing profile (bottom left) and mutant type splice variant profile (bottom right). **B** Agarose gel electrophoresis of empty pSPL3 vector (263 bp), c.1183 + 1 G > C variant (465 bp), and wild type (658 bp). **C** Sanger sequencing chromatograms of the minigene splicing assay products. Wild type (top); mutant type with skipping of exon 8 (bottom)

The potential impact of the c.1183 + 1 G > C splice site variant on mRNA splicing was examined using a minigene assay. The wild type or mutant type *GSDME* minigenes were transfected into COS7 cells, and total RNA was extracted for reverse transcription to obtain cDNA, which was amplified by PCR. Agarose gel electrophoresis of PCR amplification products showed that the wild type and the mutant type produced bands of different sizes. This yielded a 658-bp fragment in the wild type and a 465-bp fragment in the mutant type (shown in Fig. 4A, B and Additional file 1: Supplementary Fig. 1). Sanger sequencing showed skipping of exon 8 in the mutant type, which resulted in a direct connection of exon 7 to exon 9 (shown in Fig. 4A–C). This indicated that the c.1183 + 1 G > C splice site variant affected the normal splicing of exon 8, resulting in abnormal transcripts. The wild type *GSDME* contained 10 exons and encoded a protein of 496 amino acids. The skipping of *GSDME* exon 8, as a result of the variant c.1183 + 1G > C, leads to a frameshift that changes amino acid residues 331 to 371, produces a premature stop codon at position 372 and results in the loss of 125 wildtype amino acids. The target sequence was conserved throughout evolution, and the c.1183 + 1 G > C splice site variant led to the changes in the structure and function of the GSDME protein (shown in Additional file 1: Supplementary Fig. 2).

## Discussion

In 1966, Huizing et al. [13, 14] reported a five-generation Dutch family with 335 members and conducted a clinical follow-up for about 20 years [15–17]. This family had autosomal dominant deafness, with the hearing loss occurring mostly before the age of 15 years, and the minimum age of onset was 5 years. The audiometric tests revealed bilateral symmetrical sensorineural hearing loss, and the lesion site was located in the cochlea. The hearing loss started at high frequencies and deteriorated rapidly in the first three decades. Besides hearing loss, no other distinctive clinical diseases were reported, suggesting nonsyndromic hearing loss. In 1995, Van Camp et al. located the pathogenic gene to the short arm of chromosome 7 by linkage analysis [18]. In 1998, Van Laer et al. finally determined that the family’s causal variant was the c.990 + 503_990 + 1691delins132 variant in *GSDME* using Southern blot analysis and DNA sequencing [7]. The variant resulted in the skipping of exon 8 at the mRNA level and the formation of truncated GSDME protein.

Previously, 13 *GSDME* pathogenic variants and related deafness families were reported (Table 2). The phenotypes of the patients in these families were strikingly similar, being nonsyndromic, autosomal dominant, bilateral, symmetrical, postlingual onset, progressive, sensorineural hearing loss. It was accompanied with or without tinnitus, vertigo, balance disorders, and symptoms involving other vestibular systems. Hearing loss usually occurred in the first decade, initially affecting at high frequencies and progressing to severe-to-profound deafness at all frequencies rapidly in the second and third decades. In the family investigated in this study, the age of onset of hearing impairment was 18–25 years, which progressed with age. None of the members had tinnitus or vertigo, and their intelligence and speech functions were normal. The results of PTA showed severe or profound deafness in all the patients. The degree of bilateral hearing loss was about the same, and the hearing loss was mainly at high frequencies with a descending curve, implying that the hearing loss was severer as the frequency became higher. Therefore, the clinical phenotype of this family was basically consistent with that of other DFNA5 families reported in the literature.

**Table 2:** Summary of all reported *GSDME* variants leading to hearing loss

Variant DNA	Chromosomal location	Location	Effect of variant	Age of onset (year)	Hearing impairment	Ethnicity	References
c.990 + 503_990 + 1691del1189ins132	chr7:24,746,055-24747243delins	Intron 7	Skipping of exon 8	5–15	High-all frequency	Dutch	Van Laer et al. [7]
c.991-15_991-13delTTC	chr7:24,746,007–24,746,010	Intron 7	Skipping of exon 8	7–30	High frequency	Chinese	Yu et al. [29]
c.991–6 C > G	chr7:24,746,001	Intron 7	Skipping of exon 8	0–40	High-all frequency	Dutch	Bischoff et al. [30]
c.991–3 C > A	chr7:24,745,998	Intron 7	Skipping of exon 8	20–39	High-all frequency	Chinese	Wang et al. [31]
c.991–2 A > G	chr7:24,745,997	Intron 7	Skipping of exon 8	8–18	High frequency	Chinese	Chai et al. [32]
				10	High-all frequency	European	Booth et al. [33]
c.991–1 G > C	chr7:24,745,996	Intron 7	Skipping of exon 8	10–40	High-all frequency	Chinese	Yuan et al. [34]
c.1102 C > G	chr7:24,745,884	Exon 8	Skipping of exon 8	10	High frequency	European	Booth et al. [33]
c.1154 C > T	chr7:24,745,832	Exon 8	Skipping of exon 8	10	High frequency	Iranian	Booth et al. [33]
c.1158_1161delCTAC	chr7:24,745,824–24,745,828	Exon 8	Skipping of exon 8	/	/	Chinese, Italians	Ji et al. [35], Morgan et al. [36]
c.1183 G > A	chr7:24,745,803	Exon 8	Skipping of exon 8	10	High frequency	East Asian	Booth et al. [33]
c.1183 G > C	chr7:24,745,803	Exon 8	Skipping of exon 8	/	/	Chinese	Chen et al. [37]
c.1183 + 1 delG	chr7:24,745,801–24,745,802	Intron 8	Skipping of exon 8	8–30	High-all frequency	Chinese	Li-Yang et al. [38]
c.1183 + 1 G > C	chr7:24,745,802	Intron 8	Skipping of exon 8	18–25	High-all frequency	Chinese	This study
c.1183 + 4 A > G	chr7:24,745,799	Intron 8	Skipping of exon 8	11–50	High-all frequency	Chinese	Cheng et al. [39]

In this study, a novel splice site variant c.1183 + 1 G > C in intron 8 of the *GSDME* gene was identified by deafness gene NGS panel analysis in this family. The results of the minigene assay showed that the variant caused the skipping of exon 8 at the mRNA level, leading to a direct linking of exon 7 and exon 9. This variant c.1183 + 1 G > C was present in all patients, but not detected in members with normal hearing in this family. The variant was confirmed to be co‑segregating with postlingual progressive ADNSHL. Based on this, the *GSDME* c.1183 + 1 G > C was considered as the causal variant in the family.

**Fig. 5:**
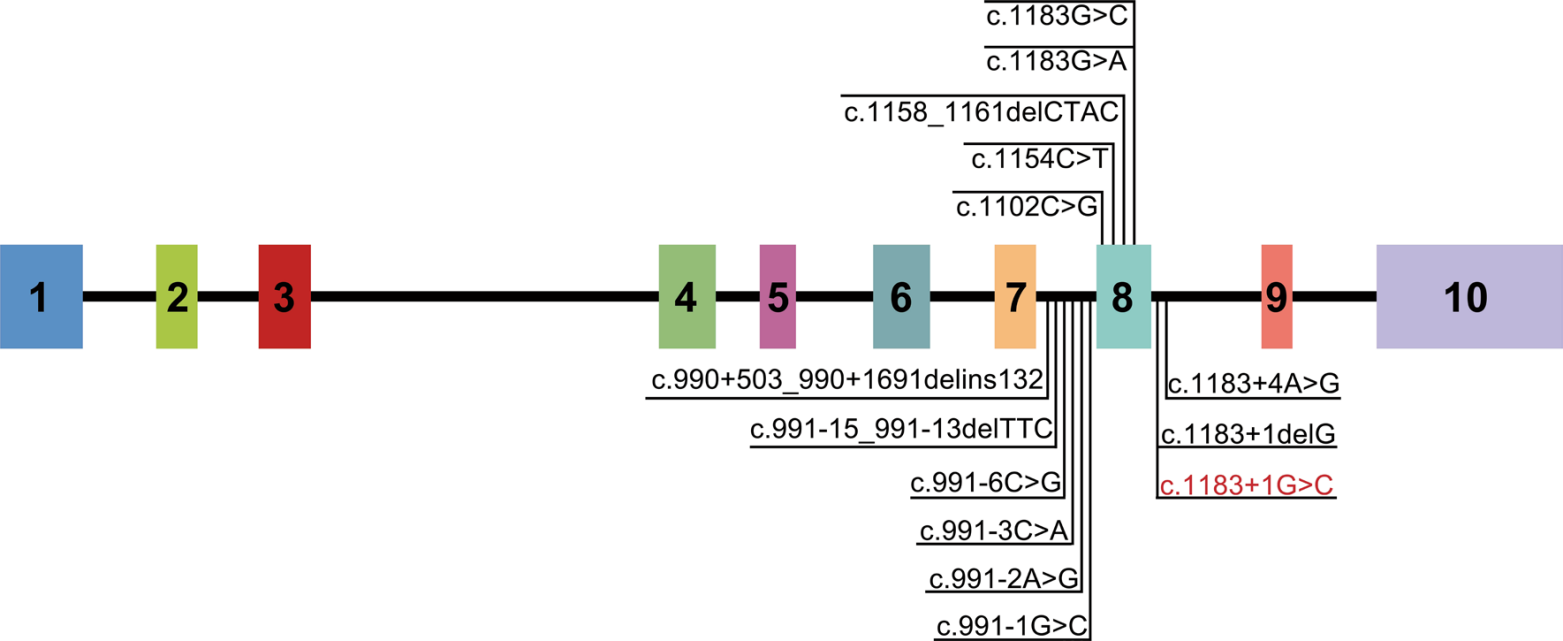
Summary of all variants in the gene *GSDME* associated with hearing loss. Indicated are the 1 novel variant in the affected individuals in the family of this study (red font) and the 13 previously described variants (black font)

To date, 13 *GSDME* variants associated with hearing loss have been recognized; 6 of them were located in intron 7, 5 of them in exon 8, and 2 of them in intron 8 (shown in Fig. 5). The *GSDME* c.1183 + 1 G > C in this study was the third variant located in intron 8. Although these variants were different at the genomic DNA level, they all eventually caused the skipping of exon 8 during splicing at the mRNA level. It was confirmed that the c.1183 + 1 G > C variant eliminated the splicing donor site on intron 8 of the *GSDME* gene, causing an abnormal splicing reaction between the intron 8 receptor site and the intron 7 donor site. This resulted in the missing transcription of exon 8. The skipping of exon 8 caused a frameshift in coding sequences. A total of 41 abnormal amino acids were translated, and a stop codon was produced at the 372–amino acid position, which eventually generated a truncated GSDME protein, missing the normal carboxyl-terminal fragment.

According to the ACMG guidelines [19, 20] and the criteria (PS3 + PM1 + PM2 + PP1 + PP4), the c.1183 + 1 G > C splice site variant is classified as ‘pathogenic’. PS3 means that ‘well-established in vitro or in vivo functional studies supportive of a damaging effect on the gene or gene product’, and minigene splicing assays used in this study is an effective technical approache to assess the impact of variants at the mRNA level. PM1 means ‘located in a mutational hot spot and/or critical and well-established functional domain without benign variation’. Even though the present novel variant leads to a frameshift, it is not a loss-of-function, but rather a gain-of-function, thus more similar to a missense variant. The novel variant and known pathogenic variants in *GSDME* are all located in and around exon 8. The variant can be analogous of a missense in a functional domain hotspot, though not the same. In addition, this variant was unequivocally consistent with the criteria PM2 (Absent from controls (or at extremely low frequency if recessive) in Exome Sequencing Project, 1000 Genomes Project, or Exome Aggregation Consortium), PP1 (Cosegregation with disease in multiple affected family members in a gene definitively known to cause the disease), and PP4 (Patient’s phenotype or family history is highly specific for a disease with a single genetic etiology). Considering the combining criteria 1 strong (PS3) and 2 moderate (PM1, PM2) and 2 supporting (PP1, PP4), the variant can be identified as ‘pathogenic’.

The molecular pathogenesis of hearing loss caused by the *GSDME* variant remains unclear. Currently, the reported pathogenic variants of *GSDME* cause exon 8 skipping at the mRNA level. A deletion or variation in other positions of *GSDME* did not cause hearing loss [21, 22], and the exon 8 knockout mice of *GSDME* had normal hearing [23]. It suggested that only the variants of *GSDME* causing exon 8 skipping could give rise to the deafness phenotype. Other studies showed that the mutant type *GSDME* could cause toxic effects such as abnormal cell morphology, cell cycle arrest, growth defects, and cell death in yeast and mammalian cells [24, 25]. These results indicated that the cytotoxicity induced by the mutant type *GSDME* might be because the truncated protein fragment expressed by exon 8 skipping acquired a new function that the wild type *GSDME* did not possess, and these variants might be a kind of 'gain-of-function’ variants.

The GSDME protein is a member of the gasdermin family, which is related to apoptosis. The GSDME protein contains N- (exon 2–6) and C- (exon 7–10) terminal domains. The hinge region (exon 6–7) is located between these two domains and connects them [26]. The apoptosis-inducing region is located in the N-terminal domain. The C-terminal domain folds back and shields the apoptosis-inducing region of the N-terminal domain to avoid inappropriate apoptotic initiation. An active caspase-3 can cleave GSDME protein at Asp270 (D270) to generate an active GSDME protein N-terminal that targets the plasma membrane to induce secondary necrosis/pyroptosis [27]. Exon 8 skipping produces a truncated protein with the shortened C-terminal domain, uncovering the apoptosis-inducing region, which may have a similar biological function as caspase-3 cleavage of GSDME protein. However, whether the hearing loss caused by mutant type *GSDME* gene is related to secondary necrosis/pyroptosis of hearing cells needs further investigation [28].

## Conclusion

Collectively, this study reported a six-generation Chinese family with ADNSHL. In this family, a novel *GSDME* splice site variant c.1183 + 1 G > C leading to the skipping of exon 8 was identified through deafness gene NGS panel analysis and co-segregation analysis. This was the third variant in *GSDME* intron 8 related to hearing loss. This finding supported the pathogenicity of the splice site variant c.1183 + 1 G > C in the *GSDME* gene. The data presented in this study extended the pathogenic variants spectrum of the *GSDME* gene, provided an important molecular interpretation and diagnosis for these patients with ADNSHL, and promoted the development of genetic counseling for inherited deafness.

## Supplementary Information

**Additional file 1**. Supplementary figure 1, 2, 3 and Supplementary table 1.

**Additional file 2.** Supplementary table 2. The list of 3255 variants in Raw Data.

**Additional file3.** Supplementary table 3. Three variants retain after the filtering.

## Data Availability

The datasets used and/or analyzed in the present study are available from the corresponding author on reasonable request. The sequencing dataset has been deposited in NCBI Sequence Read Archive, and the BioProject ID is PRJNA818115(https://www.ncbi.nlm.nih.gov/sra/PRJNA818115).
