# Supplementary material for: A novel splice site variant c.1183 + 1 G > C in DFNA5 causing autosomal dominant nonsyndromic hearing loss in a Chinese family
Source: BMC Med Genomics. 2022 Jul 21;15:163. doi: 10.1186/s12920-022-01315-8 (PMC9306051; doi:10.1186/s12920-022-01315-8)
Supplement: Supplementary file 1 — Additional file 1. Supplementary figure 1, 2, 3 and Supplementary table 1. [file 12920_2022_1315_MOESM1_ESM.docx]

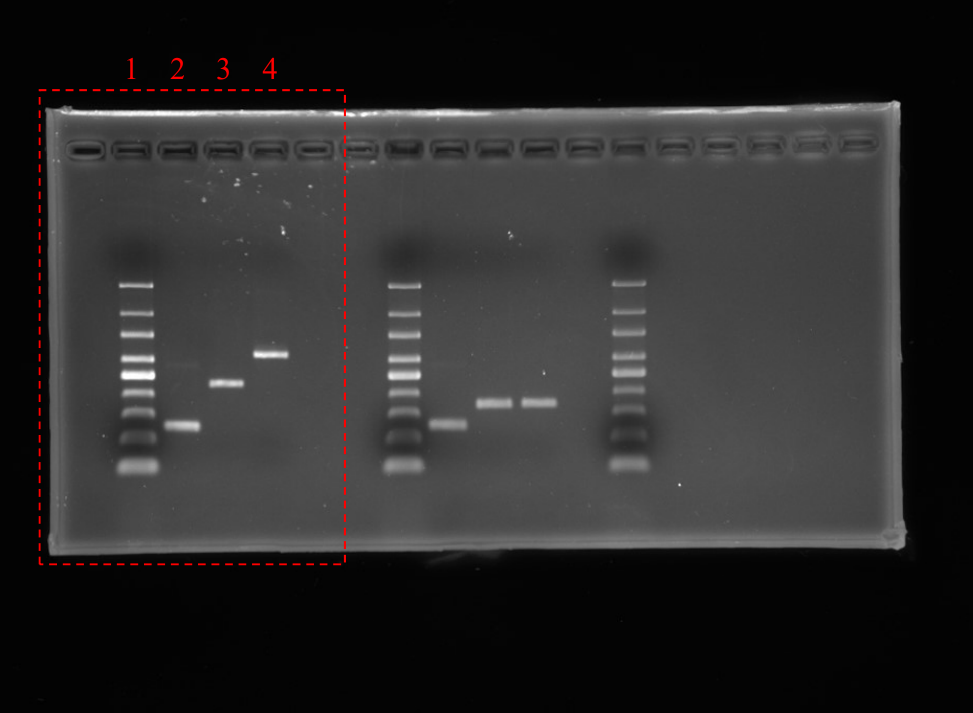


Supplementary figure 1. The full-length gels and blots of Figure 4B. The part surrounded by the red dotted line is Figure 4B. Lane 1, Marker; Lane 2, empty pSPL3 vector (263 bp), Lane 3, c.1183+1 G>C variant (465 bp); Lane 4, wild type (658 bp).


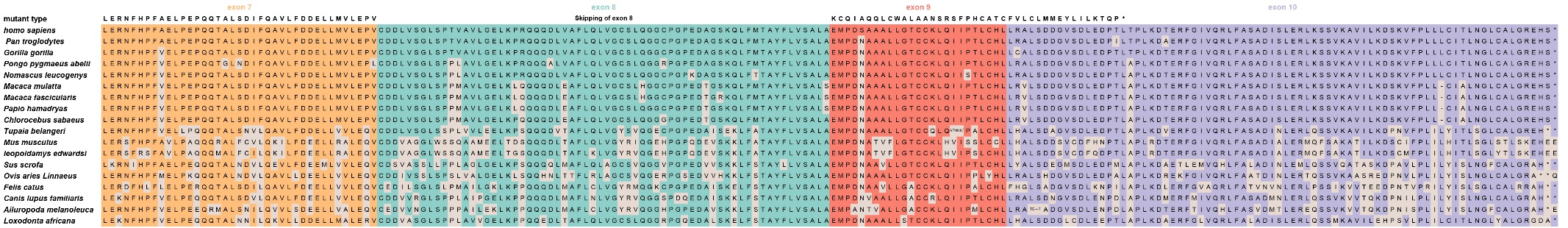


Supplementary figure 2. Conservation analysis of target amino acid residues (encoded by exon 7–exon 10) across a range of species.


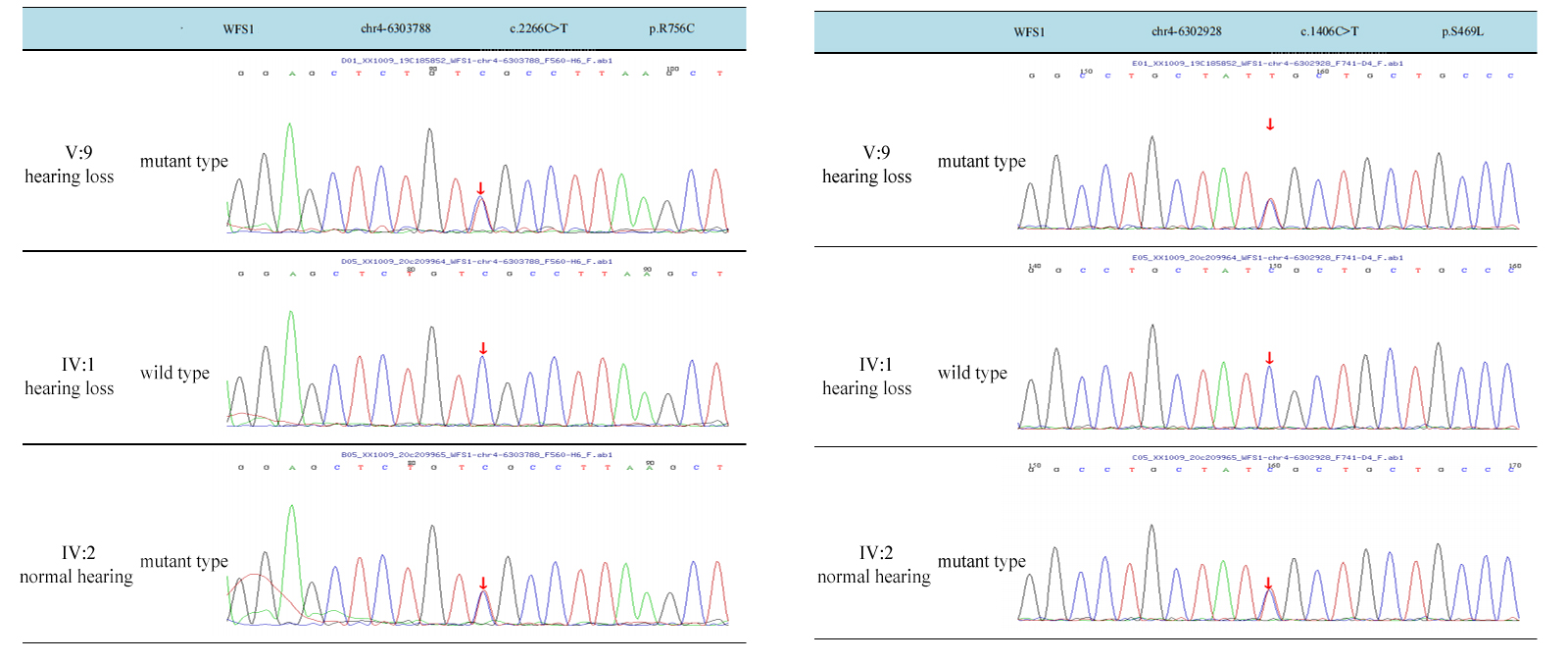


Supplementary figure 3. Sanger sequencing chromatograms of the *WFS1* c.2266C>T and 1406C>T from the participant V:9, IV:1 and IV:2. 2 variants in the WFS1 gene didn't segregated with the affected status in this family

Supplementary table 1. The list of genes that were analyzed.

| ABCD1 | ABHD12 | ABHD5 | ACO2 | ACOX1 | ACTB | ACTG1 | ADCY1 |
| --- | --- | --- | --- | --- | --- | --- | --- |
| ADGRV1 | AIFM1 | AK2 | ALMS1 | ALX3 | ALX4 | AMER1 | ANKH |
| ANKRD11 | AP1S1 | ARSE | ASPA | ATP1A3 | ATP2B2 | ATP6V1B1 | ATP6V1B2 |
| ATRX | BCAP31 | BCOR | BCS1L | BDP1 | BEAN1 | BMP1 | BRAF |
| BSND | BTD | CABP2 | CACNA1D | CATSPER2 | CCDC50 | CD151 | CD164 |
| CDC14A | CDH23 | CEACAM16 | CHD7 | CHM | CHSY1 | CIB2 | CIDEA |
| CISD2 | CLCN7 | CLCNKA | CLCNKB | CLDN14 | CLIC5 | CLPP | CLRN1 |
| COCH | COL11A1 | COL11A2 | COL1A1 | COL1A2 | COL2A1 | COL4A3 | COL4A4 |
| COL4A5 | COL4A6 | COL9A1 | COL9A2 | COLEC10 | COLEC11 | COQ6 | COX6B1 |
| CRTAP | CRYM | DCDC2 | DCHS1 | DDX11 | DHODH | DIABLO | DIAPH1 |
| DIAPH3 | DLX5 | DMP1 | DMXL2 | DNA2 | DNAJC17 | DNAJC3 | DNMT1 |
| DSPP | DVL1 | ECM1 | EDN1 | EDN3 | EDNRA | EDNRB | EFTUD2 |
| EHMT1 | ELAC2 | ELMOD3 | ENPP1 | EPS8 | EPS8L2 | ERAL1 | ERCC6 |
| ERCC8 | ESPN | ESRP1 | ESRRB | EYA1 | EYA4 | FAT4 | FGF10 |
| FGF3 | FGF8 | FGF9 | FGFR1 | FGFR2 | FGFR3 | FKBP10 | FKBP14 |
| FLNA | FLNB | FLVCR2 | FOXC1 | FOXI1 | FREM1 | FUCA1 | FXN |
| GAB1 | GALE | GATA3 | GDF3 | GDF5 | GDF6 | GFER | GIPC3 |
| GJA1 | GJB1 | GJB2 | GJB3 | GJB6 | GLYAT | GMPPA | GMPPB |
| GNAI3 | GPC3 | GPSM2 | GRHL2 | GRXCR1 | GRXCR2 | GSC | GSDME |
| GSTP1 | GSTT1 | GUCY2D | HARS | HARS2 | HGF | HMX1 | HOMER2 |
| HOXA1 | HOXA11 | HOXA2 | HOXB1 | HPD | HSD17B10 | HSD17B4 | HSPA1A |
| HSPA1L | HSPA2 | IARS2 | IDS | IFITM5 | IFNLR1 | IGF1 | IL13 |
| ILDR1 | IRX5 | ITM2B | KARS | KAT6B | KCNE1 | KCNJ10 | KCNQ1 |
| KCNQ4 | KITLG | KRT9 | LAMA3 | LARS2 | LHFPL5 | LHX3 | LMNA |
| LMX1A | LOXHD1 | LRP2 | LRP4 | LRTOMT | MAF | MAN2B1 | MANBA |
| MARVELD2 | MASP1 | MCM2 | MEOX1 | MET | MFN2 | MGP | MIR182 |
| MIR183 | MIR96 | MITF | MPZ | MPZL2 | MSRB3 | MT-RNR1 | MT-TA |
| MT-TC | MT-TE | MT-TF | MT-TH | MT-TI | MT-TK | MT-TL1 | MT-TP |
| MT-TQ | MT-TR | MT-TS1 | MT-TS2 | MT-TT | MT-TW | MYH14 | MYH9 |
| MYO15A | MYO1A | MYO1E | MYO3A | MYO6 | MYO7A | NARS2 | NDP |
| NDRG1 | NEFL | NELL2 | NF2 | NLRP3 | NOG | NOP56 | NSD1 |
| OFD1 | OPA1 | OSBPL2 | OSTM1 | OTOA | OTOF | OTOG | OTOGL |
| P2RX2 | P3H1 | PABPN1 | PAX1 | PAX2 | PAX3 | PCDH15 | PCDH9 |
| PCNA | PDE1C | PDSS1 | PDSS2 | PDZD7 | PEX1 | PEX10 | PEX11B |
| PEX12 | PEX13 | PEX14 | PEX16 | PEX19 | PEX2 | PEX26 | PEX3 |
| PEX5 | PEX6 | PEX7 | PHYH | PIGL | PJVK | PLCB4 | PLEKHM1 |
| PLOD3 | PLP1 | PMP22 | PNPLA8 | PNPT1 | POLD1 | POLG | POLG2 |
| POLR1C | POLR1D | POU3F4 | POU4F3 | PPIB | PPIP5K2 | PQBP1 | PROK2 |
| PROKR2 | PRPS1 | PRRX1 | PTPN11 | PTPRQ | PTPRR | RAB23 | RAB40AL |
| RAF1 | RDX | RECQL4 | REST | RIPOR2 | RNASEH1 | RNASET2 | ROR1 |
| RPGR | RPS6KA3 | RRM2B | S1PR2 | SALL1 | SALL4 | SCO1 | SEC23A |
| SEMA3E | SERAC1 | SERPINB6 | SERPINF1 | SERPINH1 | SETBP1 | SIX1 | SIX5 |
| SLC17A8 | SLC19A2 | SLC22A4 | SLC25A4 | SLC26A4 | SLC26A5 | SLC29A3 | SLC33A1 |
| SLC44A4 | SLC4A11 | SLC52A2 | SLC52A3 | SLC9A1 | SLITRK6 | SMAD4 | SMARCA4 |
| SMARCB1 | SMPX | SNAI2 | SNX10 | SOD1 | SOD2 | SOST | SOX10 |
| SOX2 | SOX9 | SPARC | SPTBN4 | SQSTM1 | ST3GAL5 | STRC | SUCLA2 |
| SUCLG1 | SYNE4 | TBC1D24 | TBX22 | TCIRG1 | TCOF1 | TECTA | TFAP2A |
| THOC1 | THRB | TIMM8A | TJP2 | TMC1 | TMEM126A | TMEM132E | TMIE |
| TMPRSS3 | TMPRSS4 | TNC | TNFRSF11A | TNFRSF11B | TNFSF11 | TP63 | TPRN |
| TRIOBP | TRMU | TSHZ1 | TSPEAR | TUBB4A | TUBB4B | TWIST1 | TWNK |
| TXNL4A | TYMP | TYR | UBR1 | UGT1A1 | UQCC2 | USH1C | USH1G |
| USH2A | VHL | WBP2 | WFS1 | WHRN | XPNPEP3 | YAP1 |  |
